# Caregivers and multidisciplinary team members’ perspectives on shared decision making in Duchenne muscular dystrophy: A qualitative study

**DOI:** 10.1186/s13023-025-03555-0

**Published:** 2025-03-10

**Authors:** Elise Schoefs, Thomas Desmet, Evelyn Lerinckx, Liesbeth De Waele, Sam Geuens, Conny Pelicaen, Luc Meeus, Steven Simoens, Chantal Van Audenhove, Mieke Mommen, Rosanne Janssens, Isabelle Huys

**Affiliations:** 1 Department of Pharmaceutical and Pharmacological Sciences, KU Leuven, Leuven, Belgium; 2 Healthcare Management Centre, Vlerick Business School, Ghent, Belgium; 3 Pfizer NV, Elsene, Belgium; 4 Department of Child Neurology, University Hospitals Leuven, Leuven, Belgium; 5 Department of Development and Regeneration, KU Leuven, Leuven, Belgium; 6 Duchenne Parent Project Belgium, Haacht, Belgium; 7 Department of Public Health and Primary Care, KU Leuven, Leuven, Belgium

**Keywords:** Duchenne muscular dystrophy, Interprofessional model of shared decision making, Patient decision aids, Informational needs, Multidisciplinary team, Patient engagement

## Abstract

**Background:**

As new therapies for Duchenne muscular dystrophy (DMD) are entering the market, shared decision making (SDM) will become increasingly important. Therefore, this study aimed to understand (1) Belgian stakeholders’ knowledge and perceptions of SDM in DMD treatment decision making, (2) the current state of SDM implementation in DMD in Belgium, examining the role of all involved parties, and (3) the barriers and facilitators for SDM in DMD in the Belgian context.

**Methods:**

In this qualitative study, semi-structured interviews with the multidisciplinary team (MDT) of individuals with DMD (*n* = 18) and caregivers thereof (*n* = 11) were conducted in Belgium. Qualitative data was analyzed thematically using the framework method.

**Results:**

Most caregivers were unfamiliar with the term SDM, while MDT members were aware of it but struggled to define it consistent with existing literature. Despite acknowledging some drawbacks, participants valued SDM as an important process in DMD care, noting its presence in current practice. However, both MDT members and caregivers sometimes questioned the necessity of SDM due to limited treatment options available. Consequently, decision making predominantly relied on (child) neurologists sharing information and seeking consent from caregivers and individuals with DMD for a proposed treatment. Participants highlighted the important role of the MDT, with each professional contributing its unique expertise to SDM. To reduce existing barriers and enhance the SDM process, participants called for clear and transparent information regarding different treatment options, including clinical trials, and detailed information on how treatments might affect patients’ daily life.

**Conclusion:**

This study identified an increased need for easily understandable information, particularly regarding DMD care in general, but also about clinical trials covering new and emerging therapies. Developing specific evidence-based tools could support stakeholders’ understanding of this information, thereby enhancing implementation of the SDM process in DMD care. Further, as the treatment landscape of DMD evolves, it will become increasingly important for patients to be supported by an MDT, as they can provide information on clinical trials (e.g., study coordinators), emotional support (e.g., psychologists, nurses), and decisional guidance (e.g., neurologist).

**Supplementary Information:**

The online version contains supplementary material available at 10.1186/s13023-025-03555-0.

## Introduction

Duchenne muscular dystrophy (DMD) is a rare disease caused by mutations in the *DMD* gene that encodes dystrophin, an essential protein for maintaining muscle fiber integrity and stability [1, 2]. The absence of this protein leads to the loss of stability and function of the muscle fibers in the skeletal, cardiac, and smooth muscles [2–4]. As a result, patients with DMD experience progressive muscle weakness and degeneration that leads to difficulties with movement, as well as respiratory and cardiac complications [3]. Therefore, DMD can be considered a multisystemic disease that requires management through a multidisciplinary approach [5]. In Belgium, approximately 300 DMD patients receive care from an multidisciplinary team (MDT) at one of the seven neuromuscular reference centers (NMRCs) across the country’s three regions: Flanders, Brussels, and Wallonia. These specialized centers are responsible for the care of people with neuromuscular diseases in terms of diagnosis, treatment, and counseling [6, 7]. While the specific composition and way of working of MDTs may vary among NMRCs, they generally include diverse healthcare professionals (HCPs) from different disciplines, such as a (child) neurologist, cardiologist, pulmonologist, orthopedic surgeon, physiotherapist, occupational therapist, social worker, (child) psychologist, speech therapist, nutritionist, and specialist nurse. Each team member fulfills a specific role with the collective goal of keeping patients’ quality of life as high as possible [8].

Currently, there are limited therapeutic options to treat patients with DMD, and curative therapy remains elusive. However, thanks to improved care, more individuals with DMD are now living into their early 30s and beyond [9]. The current standard of care consists of chronic corticosteroid treatment [10]. In recent years, therapeutic advancements have emerged, including technologies such as exon skipping, gene therapy, and other strategies targeting the secondary consequences of dystrophin deficiency, possibly offering promising breakthroughs for the treatment of patients with DMD [11]. Notably, multiple exon skipping therapies have been approved in the United States [12–16], whereas in Europe, only Vamorolone (Agamree^®^) has recently been granted marketing authorization [17]. Eligible patients with DMD in Europe may receive other emerging therapies through participation in clinical trials [18, 19].

Although new and emerging therapies for DMD show promising progress, their current status in clinical trials introduces uncertainties regarding safety, efficacy, and potential long-term adverse events [20]. This uncertainty is particularly pronounced in gene therapy, where risks and side effects reported during ongoing clinical trials may significantly influence patients’ choices regarding therapy options. However, there is no equivalent obligation to report benefits, which may introduce bias into the information available to patients and caregivers. This highlights the critical role of MDT members in setting realistic expectations for (experimental) treatment options based on the latest evidence available. Consequently, engaging in open dialogue with patients and their caregivers during the decision-making process is crucial. This ensures that the treatment and its effects, side effects, risks, and uncertainties align with the personal preferences and values of both patients and caregivers [21]. From an ethical standpoint and in respect to patients’ rights, shared decision making (SDM) is the most appropriate and thoroughly researched model to apply in this context, facilitating patient-centered care and informed decision making [22–24].

SDM is a collaborative conversation model wherein at least two participants, the clinician and the patient, are involved and share information. For pediatric patients or individuals requiring additional support, caregivers—such as parents or legal guardians—often also play a vital role in the process. Clinicians provide information, offer options, and describe the associated benefits and risks. Patients, and when relevant, their caregivers, process this information, consider the options and potential outcomes, and engage in dialogue to express their preferences and values, ensuring that decisions align with their unique needs and priorities. This process ensures that both participants are well informed, fostering an open discussion that culminates in a joint decision-making approach [22, 25]. Although the principles of SDM apply to many healthcare decisions, they are particularly important in preference-sensitive situations, such as in the clinical treatment decision-making phase of DMD, where uncertainties exist [26, 27]. SDM can be facilitated by patient decision aids (PtDAs), evidence-based tools in diverse formats such as leaflets and online applications that (1) make clear that a decision has to be made, (2) provide evidence-based information about treatment options, and (3) help patients clarify their preferences for these options [28]. In addition to ethical considerations for the implementation of SDM, evidence suggests that SDM not only enhances professional-patient relationships but also contributes to improved decision making and ultimately, better health outcomes [28, 29].

Despite the acknowledged importance of SDM in preference-sensitive situations and its supported evidence, its application in DMD clinical practice remains unclear, with potential barriers to its implementation yet to be revealed. Notably, existing SDM models often focus on the patient-clinician dyad. In contrast, in DMD, the SDM process is broader, including the MDT and the caregiver next to the (child) neurologist and patient [30]. However, the literature lacks insights into how various HCPs within an MDT contribute to SDM and what their specific roles in this process entail. Therefore, this study aimed to understand (1) Belgian stakeholders’ knowledge and perceptions of SDM in DMD, (2) the current state of SDM implementation in DMD in Belgium, examining the role of all involved parties, and (3) the barriers and facilitators for SDM in DMD in the Belgian context.

## Methods

### Design of the study

This qualitative study utilized semi-structured interviews with members of the MDT taking care of DMD patients within Belgian clinical practice and caregivers of individuals with DMD. Interviews were conducted between February 2023 and April 2023. Details on the methods and results of the interviews were reported according to the guidelines of Hollin et al. [31], and the consolidated criteria for reporting qualitative research (COREQ) checklist was completed (see Appendix 1) [32]. Ethical approval was obtained from the Ethical Committee Research UZ/KU Leuven in Belgium (S66893).

### Development of the study materials

The interview guides were informed by a supportive literature review with a systematic approach. Interview questions were tailored towards caregivers and the various professions of the MDT. The interview guides were reviewed by a member of the patient organization “Duchenne Parent Project Belgium” (DPP Belgium) and an SDM expert (C.V.A). An online preparatory form was created and distributed prior to the interviews. The online form contained an informed consent form and a short survey to assess participants’ demographic information. The form intended for caregivers contained additional disease-specific questions about the individual with DMD, as well as questions aimed at assessing their health status. For members of the MDT, supplementary questions related to their professional experience in treating DMD patients were included. All participants completed the online form and provided informed consent prior to their participation.

### Recruitment

Members of an MDT working in a NMRC and caregivers of individuals with DMD who speak Dutch and reside in Belgium were eligible to participate in the study. The definition of caregiver from Jimenez-Moreno et al. [33] was used in this study, stating that a caregiver is a spouse, partner, legal guardian, close relative, or other adult close to the family living either in the same house or in contact with the DMD individual in a caregiver relationship at least four times per week for at least one hour or more per day. Recruiting parties for caregivers involved the patient organization DPP Belgium and the NMRC at the University Hospitals of Leuven (UZ Leuven), which were respectively asked to distribute a recruitment flyer within their networks and in the hospital. MDT participants were recruited using purposive sampling and snowballing techniques. Dutch-speaking members of NMRCs were identified based on their specific expertise and contacted via e-mail addresses publicly available on hospital websites. The selection of MDT members for interviews was based on the input from a key opinion leader, who advised on which areas of expertise could play a role in the SDM process and should be included in the study.

### Conduct of the interviews

During the semi-structured interviews, an interview guide with predetermined questions and topics was used. However, additional questions were asked based on the topics raised during the interviews. Pre-determined topics included (1) knowledge and perceptions of SDM, (2) the current implementation of SDM and the role of all parties involved, and (3) possible barriers and facilitators to implementing SDM, including specific questions about PtDAs. Between topics, additional information was provided based on scientific and grey literature. This included explanations about SDM using the three-talk model of Glyn Elwyn et al. [34], new and emerging treatments (exon skipping, gene therapy, and other possible treatment options), as well as PtDAs. This ensured that participants gained a deeper understanding on what these concepts entailed and were prepared to address questions arising during discussions.

Participants were interviewed in their native language (Dutch) and audio recorded. Each interview lasted approximately one hour and was conducted in person or online depending on the participants’ preferences.

### Analysis

Participants’ self-reported answers from the online form were analyzed using descriptive statistics. Answers were summarized using averages and standard deviations. Health literacy was determined using Chew’s et al. [35] set of brief screening questions.

Audio recordings of the interviews were transcribed ad verbatim in the original language by E.L. and pseudonymized. Subsequently, the transcripts were subjected to the framework analysis method, a qualitative content analysis of text data in which overarching themes were developed [36]. Deductive and inductive codes were created from the interview guides and retrieved data. A final coding tree was created based on these codes and discussed between E.S., T.D., E.L., and M.M. After consensus was reached, a codebook was created (see Appendix 2), whereafter the coding tree was uploaded in NVivo and applied to all transcripts to classify sections of transcripts related to a particular theme under the respective code. All data were summarized in a framework matrix. The data of the interviews were interpreted, summarized per code, and pseudonymized quotes of individual interviewees were added for clarification.

## Results

### Participant characteristics

Initial contact was made with 22 MDT members, of whom 18 participated in the study. Reasons for not participating included (1) lack of time, (2) lack of interest to participate, or (3) no response to the invitation email. For caregivers, recruitment rates could not be determined as flyers were distributed by various parties, and the caregivers had to initiate contact with the research team. Ultimately, 11 caregivers (parents) of children with DMD reached out and participated in the study next to one individual with DMD. In total, 30 interviews were conducted, with 29 reported on. The interview with an individual with DMD was excluded due to its unique and potential diverged perspective from the caregiver-proxy viewpoints central to this study. Furthermore, excluding this interview ensured the anonymity of the participant, as there was only one individual with DMD interviewed.

Members of an MDT had an average age of 42.4 years (SD ± 11.8) and were predominantly female (83.3%). They were working in four different NMRCs across Belgium (three from Flanders and one from Brussels) and had the following professions: (child) neurologist (*n* = 5), child psychologist (*n* = 3), physiotherapist (*n* = 2), occupational therapist (*n* = 1), nurse (*n* = 1), dietician (*n* = 1), and social worker (*n* = 1). On average, they had 9.6 years of experience in DMD (range 1 to 28 years). They reported seeing an average of 36.7 patients annually (13–180) while spending an average of 48.0 min during each contact moment. Two-thirds of the participants (66.7%) were involved in academic research, while a minority (27.8%) was engaged in the development of clinical guidelines.

The children of participating caregivers were followed up in three different NMRCs in Belgium (Flanders) and were at different disease stages. They had an average age of 12.2 years (SD ± 6.2), with eight out of eleven boys relying on a wheelchair and one individual being bedridden. Most individuals with DMD (81.8%) received corticosteroid treatment. Health literacy was generally adequate for most caregivers (83.0%), with one participant exhibiting marginal health literacy and another exhibiting low health literacy. Concerning the relationship with their treating HCP, eight participants (72.7%) reported a good relationship, two (18.2%) reported a very good relationship, and one (9.1%) reported a neutral relationship. In terms of information provided on DMD by an MDT, three caregivers were neutral (27.3%), four satisfied (36.4%), and three very satisfied (27.3%). Only one caregiver expressed dissatisfaction with the information provided.

### Stakeholders’ knowledge and perceptions of SDM in DMD

Perceptions and conceptualizations of SDM varied among participants. While a few caregivers were familiar with the term, the majority expressed they had never encountered the concept before. In contrast, members of the MDT were generally acquainted with SDM. However, despite their familiarity, they had difficulties providing a definition consistent with the existing literature.

After explaining the SDM process using the tree talk model of Elwyn et al. [34], all participants recognized the significant importance of SDM and emphasized its crucial application in practice. Interviewees predominantly emphasized benefits rather than drawbacks when discussing the concept and application of SDM in practice (Table 1).

**Table 1:** Benefits and drawbacks of SDM according to participants. The stakeholder group that mentioned each statement during the interviews is indicated between brackets. DMD; Duchenne muscular dystrophy, HCP; Healthcare professional, MDT; Multidisciplinary team, SDM; Shared decision making

Opinions	Illustrative quote
**Benefits of SDM**	
1. Can inform individuals with DMD and caregivers about all available options including advantages, disadvantages, risks, and uncertainties. (Members of the MDT, caregivers)	*“I think being informed about everything should also provide a sort of reassurance*,* in my opinion.” (MDT_14)*
2. Can give individuals with DMD and caregivers the feeling of being heard, ensuring that their opinion is taken into account when making decisions. (Members of the MDT, caregivers)	*“That you have the feeling that you are being heard. After all*,* it is about your child or about*,* well*,* the patient themselves. It’s quite important that you are allowed to say something about your own life or about your child’s life because these are often significant matters that you would like to be involved in the decision-making process*,* I think. Or at least have a say in*,* let’s put it that way.” (Caregiver_8)*
3. Can ensure individuals with DMD and caregivers that decisions are made with their support. (Members of the MDT, caregivers)	*“I believe the decision that is then made is indeed fully supported*,* or at the very least*,* the parents or the patient are well aware of all the potential options*,* making it a conscious choice.” (MDT_15)*
4. Can ensure support from all the members of the multidisciplinary team when a decision is being made. (Caregivers)	*“I believe that when a decision is made within a team*,* perhaps by two or three physicians and a treating physiotherapist*,* along with us and the patient it is the most thoroughly considered approach*,* and that it is not just one person who makes a decision about a treatment.” (Caregiver_1)*
5. Can establish a long-term bond of trust between individuals with DMD, caregivers, and HCPs. (Members of the MDT)	*“I do find it important that you can build a connection in some way. And I think shared * *decision making really forms a part of the foundation for building that connection.” (MDT_7)*
6. Can positively impact therapy compliance. (Members of the MDT)	“*If caregivers do not agree with the proposed treatment but are still required to follow it*,* it often leads to discontinuation of the treatment or seeking alternative options*,* which may not always be in the best interest of the child.” (MDT_17)*
**Drawbacks of SDM**	
1. Can overwhelm caregivers by providing too much information. (Members of the MDT, caregivers)	*“Offering an excessive amount of information might overwhelm certain individuals with an overflow of details.” (Caregiver_9)*
2. Can be a time-consuming process that needs extra effort. However, applying SDM can have long-term benefits. (Members of the MDT, caregivers)	*“I don’t see that as a disadvantage*,* because I think that it can bring extra benefits later if done in the right way. It can prevent later discussions or problems.” (MDT_14)*

All participants considered the involvement of caregivers and individuals with DMD in treatment decisions as inherently understood and of utmost importance, given its profound impact on their lives: *“I find it only reasonable that in the case of such a drastic investigation… it should be up to the parents to make the final decision.”* (Caregiver_2) and “*I believe that having a say in what you do is simply one of the human rights in any society.”* (MDT_6). Consistent with the key concepts of SDM, some MDT members emphasized the importance of providing caregivers and individuals with DMD with sufficient information and resources to be actively involved in the decision-making process. Participants also collectively agreed that a key step in the SDM process is asking caregivers and individuals with DMD if they wish to be involved in the decision making itself. They stressed the equal importance of respecting the wishes of those who choose not to be involved, highlighting the need for a thoughtful conversation to understand their reasons for non-involvement.

### Current implementation of SDM and the role of all involved parties

Members of an MDT mentioned that SDM is frequently applied in their practice, even though it may not always be explicitly expressed as such: *“I think that I do it that way*,* or at least I try to*,* but I don’t consciously have that particular theory in mind. Nonetheless*,* I believe that I do follow the principles of SDM in my practice.”* (MDT_14). They also recognized an evolution in their approach, acknowledging that in the past, treatment decisions were often unilateral without considering patients’ preferences. Presently, while HCPs may express a preference for a particular treatment or suggest a clinical study, they emphasize the importance of seeking caregivers’ approval after explaining the options, ultimately leaving the decision to the individual with DMD and their caregiver. They further mentioned that it is not common practice to ask patients explicitly if they desire a certain treatment over another. Instead, HCPs generally attempt to explain the necessity to initiate a particular treatment, with caregivers placing trust in the HCP to offer the right information and to make the correct recommendations.

Caregivers generally felt included and supported in the decision-making process, expressing they had a say in the treatment plan: *“We always felt that the final decision was in our hands.”* (Caregiver_2). However, instances of perceived exclusion were noted. This could be attributed to the limited awareness of the concept of SDM; patients did not always view every decision-making moment as a collaborative choice with their HCP, especially when choices were presented without explicitly mentioning the need for an SDM process. For example, according to caregivers’ perceptions, corticosteroid treatment is often initiated without inquiry about the patients’ and caregivers’ preferences. Although considered the standard of care and the best option at the time, caregivers desired information about the consequences of this choice for their child, as well as the ramifications if one chooses not to opt for the treatment. Additionally, despite being a key concept of SDM, participants mentioned that the option of not undergoing treatment was not always explicitly discussed, resulting in decisions about which therapy to take rather than refraining from therapy: *“They are convinced that this is the best way to help the patient*,* but sometimes they overlook the fact that there are also people who might not want to be helped.”* (Caregiver_3).

Regarding clinical studies, some caregivers felt inadequately informed about ongoing clinical trials and expressed a desire for more information, even about those trials their child was ineligible for. Most MDT members confirmed this perspective, noting that they assess clinical studies for each patient, potentially reducing the need to present certain studies to caregivers and individuals with DMD. However, most caregivers preferred explicit discussions about all clinical studies during consultations, including those not recommended or suited for their child, with explanations. They viewed this as essential to SDM, preventing confusion from external information sources and ensuring a comprehensive understanding of all available options.

#### Role of the individual with DMD and caregiver in the SDM process

While there is a common perception that individuals with DMD may be too young to actively participate in the decision-making process, caregivers expressed the importance of informing their child at a certain age with appropriate information that they can comprehend. The MDT also underscored the significance of involving individuals with DMD in decisions from a certain (non-specified) age onward, emphasizing the delivery of information tailored to the understanding of those under fourteen years rather than direct participation. Notably, a non-treating HCP raised the pertinent issue of balancing parental opinions with the child’s input, acknowledging the challenge when the perspectives of the child and the parents diverge: *“I believe that parents have the right to speak on behalf of their child*,* as also determined by law. However*,* as a healthcare provider*,* it can be challenging when you feel that the opinions of the child and the parents do not align.* (MDT_6).

#### Role of the MDT in the SDM process

Within the MDT, distinct levels of involvement in the decision-making process were identified, with roles being categorized as leading, supporting, informative, or limited (Table 2). While acknowledging the potential evolution of current roles to encompass new responsibilities associated with emerging treatments in the future, members of the MDT generally anticipated that their roles would remain largely consistent. Nevertheless, one treating HCP mentioned the importance of increased follow-up and communication among team members to ensure optimal patient care with these new therapies.

**Table 2:** Different roles in the SDM process of the MDT involved in the care of individuals with DMD

	Leading role	Supportive role	Informative role	Limited role
**Specialty**	Neurologist	- Psychologist - Nurse	- Study coordinator - Physiotherapist	- Social worker - Occupational therapist - Dietitian
**Task**	Primary responsibility to inform caregivers/patients, while also ensuring that the rest of the healthcare team is well-informed and able to address any additional questions or concerns caregivers/patients may raise.	An intermediary between the neurologist and the patient, facilitating the transmission of questions. Exploring underlying motivations and ensuring that patients and caregivers fully comprehend all information presented.	Provision of additional information on clinical trials or physical aspects of treatments.	Have a rather limited influence or involvement in the decision-making process.
**Illustrative quote**	*“I am responsible for informing patients. I believe that is my task. And the rest of the team should be well informed so that they can handle other additional questions because sometimes questions also come their way. They should be able to handle those and bounce them back to me if there’s something they can’t answer.” (MDT_14)*	*“I do think it’s my role*,* on one hand*,* to uncover the motivation behind their choice*,* but on the other hand*,* to also verify whether they have truly understood everything the physician explained.” (MDT_3)*	*“Often before starting a treatment*,* they reach out to me to thoroughly discuss all the specifics*,* particularly focusing on practical aspects. However*,* the physician is the one who explains the mechanism of action of that treatment.” (MDT_12)*	*“I see that happening here in our clinic*,* our physician always engages in conversations with the patients. I often see the physician consulting with study nurses and the physiotherapists.”… “I see it happening regularly. But I*,* myself*,* am not really involved in that.” (MDT_8)*

### Factors influencing the implementation of SDM in DMD practice

During the interviews, a diverse array of barriers to the implementation of SDM in DMD clinical practice were discussed next to strategies to enhance the SDM process. While certain barriers and facilitators were highlighted more prominently by specific stakeholders, multiple factors were consistently identified across both stakeholder groups (Table 3).

**Table 3:** Barriers and facilitators of SDM from the perspectives of caregivers and the MDT categorized in different levels defined by the Ottawa Model of Research Use (OMRU) [37]. DMD; Duchenne muscular dystrophy, MDT; Multidisciplinary team

	Viewpoint	Barriers	Facilitators
**Decision level**	Common	- Lack of treatment options - Fast decisions - Lack of evidence-based information regarding therapies in clinical trials	- Having enough time to make an important decision
	Members of an MDT		- Unambiguous decision making - Space for questions during the consultation - Presentation of a total treatment plan
**Adopter level**	Common	Related to caregiver/individual with DMD: - Low health literacy - Speaking another (native) language than the MDT - Forgetting or not understanding information	Related to caregiver/individual with DMD: - Professional translator available during consultations - Repetition of information to caregivers/individual with DMD during follow-up consultations
	Caregivers	Self-related: - Unable to make a decision - Emotional involvement	
	Members of an MDT	Self-related: - Difficulties in transferring medical information in a lay language Related to caregiver/individual with DMD: - Fear of making a decision - Does not want to be responsible	Related to caregiver/individual with DMD: - Empowered patients
**Relational level**	Common	- Lack of confidence/trust from the caregiver/individual with DMD in the MDT - Disagreement between caregiver(s)/individual with DMD and the MDT - Bad cooperation and communication between members of the MDT	- Confidence/trust in the MDT - Honest, transparent conversations between all parties involved
	Caregivers	- Caregiver/individual with DMD having fear of sharing their opinion - Disagreement between members of the MDT regarding the best way forward for the individual with DMD	- Caregiver/individual with DMD feeling supported and understood in their choices - MDT taking into account the needs and preferences of the caregiver/individual with DMD - Good collaboration between different hospitals wherein individual with DMD is treated - Good collaboration between MDT and home nursing
**Environmental level**	Common	- Time shortage	
	Caregivers		- The presence of a psychologist
	Members of an MDT	- Limited availability of medicines (e.g., certain therapies not being reimbursed for individuals with DMD) - Caregivers/individuals with DMD being overwhelmed by the hospital setting	- The use of interpreters - Communication skills training for members of the MDT - No sanctions for longer consultations
**Innovation level**	Common	- Lack of unbiased patient material that can support the decision-making process	- Availability of patient decision aids or other visual supportive information
	Caregivers		- Summary of information provided during the consultation - Contact person for caregiver/individual with DMD who is available to answer questions outside the hospital

#### Addressing informational needs in DMD treatment decision making

Caregivers harbored a multitude of questions regarding various aspects of new and emerging therapies for DMD, as outlined in Box 1. Recognizing and addressing these informational needs could potentially serve as a facilitator for SDM.

**Box 1:** Questions raised by caregivers on new and emerging therapies

- Is my child eligible for a study?	
- Is there any progress in gene therapy?	
- Is there an ongoing study in gene therapy?	
- Why is a viral vector needed for gene therapy?	
- Can you perform exon skipping on a deletion as well as a duplication?	
- What is the status of other medications that would be useful in the long run?	

While caregivers generally felt adequately informed about DMD, they expressed a desire for more in-depth information on clinical trials available for their child: “*We know very little about the various possibilities in studies unless you specifically look for information yourself and then ask for information about a particular study*,* but then we also notice that the knowledge about this is limited.”* (Caregiver_12). However, some members of the MDT believed that presenting all information and subsequently removing it due to ineligibility could be overwhelming and confronting for caregivers or individuals with DMD, especially when there are limited treatment options available: “*That is the reality for most patients*,* that they have no options or only one option available”… “Only a few patients have the luxury of choosing from a range of treatments.”* (MDT_11). In contrast, one treating HCP favored discussing every treatment option and clinical study, explaining why certain options might not be feasible for a particular patient, and aiming to address potential unanswered questions that individuals with DMD or caregivers might have in the future.

Both a treating HCP and caregiver suggested that creating a specialized platform featuring all clinical studies related to DMD in Belgium presented in the native language of the caregivers or individuals with DMD would be of added value. This platform could address some of the existing challenges such as the difficulty of accessing the information on the inclusion/exclusion criteria for patients, and essential information HCPs need to ask and obtain from specialized centers. A specialized platform could expedite information retrieval and streamline communication with various hospitals that are not conducting clinical trials, ensuring they do not fall behind.

#### Need for patient decision aids

Although participants were initially unfamiliar with the concept of PtDAs before its introduction in the interviews, the overall sentiment was positive while being critical, with many recognizing the role these tools could play in facilitating the SDM process when new innovative therapies reach the market. The creation of a PtDA specifically for DMD was therefore seen as a potential strategy to enhance SDM, as it can increase the understanding and engagement of both caregivers and individuals with DMD in the decision-making process, ultimately fostering increased patient-centered care. A treating HCP also expressed the belief that a PtDA could make the decision-making process more explicit and encourage people to take responsibility for their choices: *“The idea of shared decision making*,* we carry out*,* but we don’t actually do it that explicitly at all and a shared decision tool would make it much more explicit.”* (MDT_15). Further, participants also highlighted the advantage of PtDAs in consolidating all discussed information from a consultation in one accessible place, addressing the issue of information loss during appointments.

Some members of the MDT were hesitant towards the use of PtDAs, outlining specific preconditions that needed to be met before integrating them into their practice. For example, a treating HCP emphasized the critical need to meticulously organize evidence-based information within the PtDA. They also highlighted the significance of a straightforward design for the value clarification exercise (the exercise that helps patients to reveal their preferences for treatment and disease-related characteristics) to prevent confusion: “*If you can put in the nuances that are needed*,* this can definitely be a good support tool.”* (MDT_14). This HCP further highlighted that individuals may require guidance from HCPs to navigate PtDAs effectively, underlining the fact that not everyone can independently engage with the tool.

## Discussion

Although participants believed SDM was integrated into current practice, they observed that the option of no treatment was rarely discussed and that multiple treatment options were uncommon due to a lack of available treatments. Consequently, neurologists typically presented a single treatment option and sought consent from caregivers and individuals with DMD. Significant barriers to the integration of SDM included the scarcity of treatment options, the emotional challenges of making treatment decisions, and the absence of unbiased, evidence-based information on clinical trials. The study also highlighted the essential role of each MDT member, having their unique expertise to support caregivers and individuals in the SDM process.

### Optimizing the implementation of SDM via tailored information disclosure

Aligning with literature on pediatric diseases [38], our findings show that caregivers value participation in medical decisions affecting their children’s care. MDT members also acknowledged the critical role of caregivers in the decision-making process and, consistent with Shay et al. [29], believed that SDM is already integrated into their practice. However, the interviews revealed that the option to refrain from any therapy is often disregarded during consultations. Additionally, due to the limited treatment options available, caregivers often lacked awareness that a decision needed to be made. Typically, only one treatment is presented and explained by the neurologist, who then seeks consent, as noted by Karnieli-Miller et al. [39], who observed that physicians tend to use persuasive strategies to align patients’ decisions with their recommendations, rather than providing a comprehensive explanation why certain options are not recommended or available. These results align with broader research indicating that HCPs frequently do not apply the full spectrum of SDM concepts in practice [40, 41]. To uphold SDM principles, it is crucial to inquire whether families prefer information on all approved and experimental treatments or solely on the ones for which the individual with DMD is eligible. This approach allows for the provision of information on all available options, while clearly explaining why certain options are not considered suitable for the patient. Such an approach minimizes potential confusion arising from external sources such as social media and testimonials from other families, while honoring caregivers’ preferences regarding information.

### Caregivers want to protect individuals with DMD emotionally from the decision-making process

Interviews revealed that both caregivers and MDT members often perceive children with DMD as too young to be involved in decision making, consistent with the findings of Boland et al. [37]. Given that DMD primarily affects young boys, caregivers typically assume the responsibility of making decisions on their behalf. This stance, however, is not always driven by doubts about the child’s capacity for SDM but could be explained by a desire to shield them from the emotional burden of understanding the disease’s progression. Caregivers aim to protect their children from the severe psychological impact of anticipating a potentially bleak future, thereby prioritizing their child’s mental well-being and striving to provide the most carefree childhood possible. This protective inclination should be acknowledged and addressed in discussions between HCPs and caregivers, balancing the pros and cons of open communication versus protection. Such conversations are crucial, as they can determine the extent to which children with DMD might take on a more active role in the decision-making process.

Our findings also underscore the complexity of determining the appropriate age for actively including patients with DMD in medical decision making. Decision-making competence is influenced by contextual and developmental factors, such as the onset of adolescence, cognitive abilities, and maturity. Additionally, societal beliefs about competence are reflected in medical laws and regulations, further complicating this determination. Grootens-Wiegers et al. [42] highlight how brain development and individual maturity necessitate a tailored approach to decision making, while also emphasizing the importance of ethical principles and regulatory frameworks. Future research is essential to deepen our understanding of these dynamics and provide clearer guidance for integrating individuals with DMD in SDM at appropriate stages.

### The role of MDT members in SDM may evolve in the future

The interviews underscore the integral role of MDT members in SDM within NMRCs in Belgium for DMD care. This aligns with the interprofessional model of shared decision making (IP-SDM) from Légaré et al. [30] emphasizing the need for collaboration of at least two interdisciplinary HCPs in SDM to foster holistic medical, emotional, and social support. For instance, MDTs involved in DMD care play critical roles in providing evidence-based information and emotional support, with team members serving as trusted confidants for patients to openly discuss personal concerns and feelings [5, 43]. Further, the interviews revealed the invaluable contributions of each team member to the well-being of the patients, each drawing upon their specialized knowledge and unique expertise to address a wide range of patient and caregiver needs. This ranges from psychologists providing emotional support to personal struggles to social assistants providing guidance on financial matters. This collaborative approach not only supports individuals with DMD but also empowers caregivers by addressing a spectrum of needs. Furthermore, the interviews revealed that in the NMRCs close interaction between the various HCPs is facilitated on a structured basis, allowing for alignment in the care of patients on a case-by-case basis. This collaboration between HCPs is strongly applauded and is a necessary aspect of adopting the IP-SDM model.

Nevertheless, discrepancies persist among team members regarding their roles in SDM. To optimize SDM in DMD care, it is crucial to clarify and align current and future responsibilities within the SDM model. With anticipated advancements in DMD therapies, MDT roles may evolve to encompass new responsibilities. For example, study coordinators may need to translate complex clinical information into easily understandable language tailored to the health literacy levels of individuals with DMD and their caregivers. Therefore, establishing and maintaining comprehensive MDTs within NMRCs, comprising a diverse range of specialists, is essential. This approach not only maximizes SDM opportunities but also ensures continued delivery of personalized care amid evolving treatment landscapes. The way Belgian NMRCs are organized with their MDTs can further be an example for other European countries to meet the specific needs of this population.

### Overcoming barriers related to SDM

A primary barrier to SDM, as also noted by Boland et al. [37], is the restricted array of treatment choices, which can foster a perception that SDM is redundant. Our study similarly found that caregivers often viewed the decision to use corticosteroids as a predetermined standard of care set by the HCP. However, with the potential emergence of new therapy options, it will be crucial to present all available choices to individuals with DMD and their caregivers, including the option to refrain from treatment as suggested by the SDM model, thereby fostering active participation in the decision-making process. One way to facilitate this process, is via the use of evidence-based information tools such as PtDAs. However, based on the interviews, no PtDAs are currently used in Belgian clinical practice for DMD. Instead of creating entirely new tools, it is key to translate and adapt existing PtDAs developed by patient organizations or other stakeholders to align with the Belgian clinical context. This approach minimizes redundancy, leverages the wealth of existing knowledge, and ensures tools are culturally and practically relevant. As new therapies become available, the need for these tools will become increasingly essential to support effective SDM and enhance the quality of care. In the absence of such tools, we recommend that hospitals begin integrating SDM principles into consultations, even when choices are limited. This proactive approach can familiarize HCPs with SDM principles in a broader context, preparing them for future scenarios with expanded treatment options.

Furthermore, all participants emphasized the need for adequate time to make well-informed decisions and repeatedly receive tailored, evidence-based information. This aligns with findings from Chan et al. [44], highlighting the value of reviewing and revisiting medical information until fully understood. Concerns about the potentially time-consuming nature of SDM, identified as a significant environmental barrier by Légaré et al. [45] and Alsulamy et al. [46], was also raised by some interviewees. However, one treating HCP believed that implementing SDM could ultimately save time by pre-empting future discussions or issues. This perspective resonates with research by Søndergaard et al. [47], which indicated that SDM does not increase overall consultation time. To enhance SDM implementation, it is essential for MDTs to be educated about the benefits of SDM, dispelling any misconceptions about its time-intensive nature.

Participants in this study highlighted trust as a critical factor influencing the relationship between individuals with DMD and/or caregivers and HCPs. They emphasized the importance of honest and transparent conversations, feeling supported and understood in their decisions as key facilitators for SDM. This aligns with findings by Alsulamy et al. [46], who underscored the clinician-patient relationship quality as pivotal for SDM. One way to strengthen this bond of trust is to create clear, comprehensible information and transparent communication on treatment options and clinical trials, addressing caregivers’ conviction that these trials could benefit their child. To support HCPs in this endeavor, specialized platforms, such as clinicaltrial.be, listing ongoing DMD-related clinical trials in Belgium could effectively inform caregivers and individuals with DMD in monitoring trial progress and outcomes.

### Strengths and limitations of the study

Careful sampling was performed to include a diverse group of caregivers next to MDT members working in different NMRCs across Belgium. This safeguarded the confidentiality of participants and ensured a mixed group of participants with different experiences and views regarding SDM in our study. Further, a standardized interview guide which was reviewed by the patient organization DPP Belgium and an SDM expert was used during all the interviews. This ensured comprehensibility and relevance to caregivers, enhanced the validity and reliability of the study findings, and minimized potential bias. Furthermore, by using semi-structured interviews, we were able to obtain open-ended responses from individuals directly impacted by DMD, while maintaining flexibility to explore emerging themes. This approach provided in-depth insights and richer data compared to closed-ended questions or quantitative research.

This study also has limitations. First, participants who were interested in SDM and had overall more positive perceptions might have been more inclined to participate in the interviews, potentially biasing the results to more favorable attitudes toward SDM. However, the interviews also included participants with critical views on SDM. Additionally, harder-to-reach caregivers might not have been included. A notable limitation is the exclusion of individuals with DMD from the analysis. While one interview was conducted with an individual with DMD, the results were excluded to preserve the participant’s anonymity and because the insights would not have been representative of the broader population. Consequently, the perspectives of individuals with DMD, who are key stakeholders in the SDM process, were not directly included. While caregivers offer valuable insights and often serve as proxies, this study cannot address how individuals with DMD prefer to engage in decision making, the roles they wish to assume, or the specific barriers they may face in SDM. Further, representation from certain disciplines within the MDT was limited. Moreover, only Dutch-speaking participants from the Flemish and Brussels regions were included. Therefore, it cannot be determined if the findings may be extrapolated beyond these regions.

### Future research

Future research should focus on evaluating and implementing the recommendations derived from this study to enhance the adoption of SDM in the DMD care pathway. Specifically, key areas for further research and implementation include (1) investigating how individuals with DMD prefer to be involved in the decision-making process, identifying perceived barriers to their involvement, and examining how willingness to participate may vary across different disease stages, (2) determining the appropriate age, developmental stage, and skills that patients with DMD should possess to actively engage in SDM, while considering factors such as cognitive development and maturity, (3) investigating how existing and constantly developing clinical trial databases, such as clinicaltrial.be, can facilitate transparency and accessibility of ongoing clinical trials and related information, ensuring easy access for individuals with DMD, caregivers, and HCPs, (4) translating, adapting, and/or evaluating pre-existing evidence-based tools such as PtDAs to improve knowledge transfer from HCPs to individuals with DMD and caregivers, thereby facilitating SDM, and (5) strengthening the role of various MDT members, including nurses, social workers, psychologists, etc. as a direct entry point for individuals with DMD and caregivers, thereby enhancing their relationship and promoting a more integrated approach to SDM.

## Conclusion

This research shows an overall positive perception of SDM in DMD care. While participants felt that SDM was already integrated into clinical practice, corticosteroid therapy is typically presented as the standard of care and the option of refraining from any treatment is seldom discussed. The primary barrier to SDM is the limited number of treatment options available. As the treatment landscape evolves, clear, comprehensible information about DMD, clinical trials, and new therapies will be needed. Developing evidence-based tools to provide this information can support individuals with DMD and caregivers in becoming active participants in the decision-making process. The introduction of new therapies will also impact the role of the MDT, which will become more critical as they can provide information on clinical trials (e.g., study coordinators), emotional support (e.g., psychologists, nurses), and decisional guidance (e.g., neurologist).

## Electronic supplementary material

Below is the link to the electronic supplementary material.

Supplementary Material 1

Supplementary Material 2

## Data Availability

The datasets presented in this article are not publicly available because they contain information that could compromise participants’ privacy and consent. Requests to access the datasets should be directed to contact.isabellehuys@kuleuven.be.

## Abbreviations

COREQ: Consolidated criteria for reporting qualitative research
DMD: Duchenne muscular dystrophy
DPP: Duchenne Parent Project Belgium
HCP: Healthcare professional
IP-SDM: Interprofessional model of shared decision making
MDT: Multidisciplinary team
NMRC: Neuromuscular Reference Center
OMRU: Ottawa Model of Research Use
PtDA: Patient decision aid
SDM: Shared decision making
UZ: University Hospitals of Leuven
