# Supplementary material for: Caregivers and multidisciplinary team members’ perspectives on shared decision making in Duchenne muscular dystrophy: A qualitative study
Source: Orphanet J Rare Dis. 2025 Mar 10;20:113. doi: 10.1186/s13023-025-03555-0 (PMC11895160; doi:10.1186/s13023-025-03555-0)
Supplement: Supplementary file 1 — Supplementary Material 1 [file 13023_2025_3555_MOESM1_ESM.docx]

**Appendix 1: The COREQ checklist**

Consolidated Criteria for Reporting Qualitative Studies (COREQ): 32-item Checklist

| **No** | **Guide question/description** | **Response** |
| --- | --- | --- |
| Domain 1: Research team and reflexivity | | |
| *Personal characteristics* | | |
| 1. | Which author/s conducted the interview or focus group? | ES, TD, and EL |
| 2. | What were the researcher’s credentials? E.g. PhD, MD | ES and EL hold a MSc in biomedical sciences. TD holds a MSc in pharmaceutical sciences. |
| 3. | What was their occupation at the time of the study? | ES and TD are completing their PhDs in the regulatory sciences research unit of the University of Leuven. EL was a master student biomedical sciences at the time the interviews were performed. |
| 4. | Was the researcher male or female? | ES and EL are female. TD is male. |
| 5. | What experience or training did the researcher have? | ES and TD had previous experience and training in completing qualitative interviews and has trained EL via preparatory meetings and supervision during the first interviews. |
| *Relationship with participants* | | |
| 6. | Was a relationship established prior to study commencement? | No, study participants were only contacted through email or telephone by the researchers beforehand to agree on a place and time for the interview. |
| 7. | What did the participants know about the researcher? e.g. personal goals, reasons for doing the research | The participants were informed on the goal of the research and the future use of the data. |
| 8. | What characteristics were reported about the inter viewer/facilitator?  e.g. Bias, assumptions, reasons and interests in the research topic | The researchers’ background and the fact that the study was part of the masters’ thesis of EL. |
| Domain 2: Study design | | |
| *Theoretical framework* | | |
| 9. | What methodological orientation was stated to underpin the study? e.g. grounded theory, discourse analysis, ethnography, phenomenology,  content analysis | Qualitative research evaluated through framework method content analysis. |
| *Participant selection* | | |
| 10. | How were participants selected? e.g. purposive, convenience,  consecutive, snowball | Via purposive sampling and snowballing techniques. |
| 11. | How were participants approached? e.g. face-to-face, telephone, mail,  email | Via email, face-to-face contact, or social media. After initial interest, participants were contacted by EL via email or telephone to schedule the interview. |
| 12. | How many participants were in the study? | 30 participants were interviewed. |
| 13. | How many people refused to participate or dropped out? Reasons? | First contact was established with 36 possible participants of which 6 did not respond to further emails. Reasons are unknown. |
| *Setting* | | |
| 14. | Where was the data collected? e.g. home, clinic, workplace | At the hospital participants were working, at participants’ homes, or online. |
| 15. | Was anyone else present besides the participants and researchers? | With 1 patient interview the caregiver was present. For the other interviews, only the participant and one or two researchers were present. |
| 16. | What are the important characteristics of the sample? e.g. demographic data, date | See 3.1. Participants characteristics. |
| *Data collection* | | |
| 17. | Were questions, prompts, guides provided by the authors? Was it pilot  tested? | Yes, a full interview guide was prepared. This guide was validated through input from a member of the patient organization Duchenne Parent Project and an academic expert in SDM. The interview guide was tested in 3 interviews. As no major modifications were deemed necessary, these interviews were included in the analysis. |
| 18. | Were repeat interviews carried out? If yes, how many? | No. |
| 19. | Did the research use audio or visual recording to collect the data? | Audio recording was used to collect the data. |
| 20. | Were field notes made during and/or after the interview or focus group? | All audio recordings were fully transcribed and field notes were taken. |
| 21. | What was the duration of the inter views or focus group? | The total duration of interviews was between 25 and 90 minutes. |
| 22. | Was data saturation discussed? | Data saturation was discussed between the researchers of the study. |
| 23. | Were transcripts returned to participants for comment and/or correction? | No. |
| Domain 3: analysis and findings | | |
| *Data analysis* | | |
| 24. | How many data coders coded the data? | EL coded all the transcripts. |
| 25. | Did authors provide a description of the coding tree? | Yes, a codebook was developed including a definition of the code, the inclusion and exclusion criteria and an example per code. |
| 26. | Were themes identified in advance or derived from the data? | Both deductive and inductive codes were used. |
| 27. | What software, if applicable, was used to manage the data? | NVivo was used to manage the data. |
| 28. | Did participants provide feedback on the findings? | No. |
| *Reporting* | | |
| 29. | Were participant quotations presented to illustrate the themes/findings?  Was each quotation identified? e.g. participant number | Yes, quotes are followed by the participant code. |
| 30. | Was there consistency between the data presented and the findings? | Yes. |
| 31. | Were major themes clearly presented in the findings? | Yes, all major themes related to shared decision making have been discussed. |
| 32. | Is there a description of diverse cases or discussion of minor themes? | Yes, attention was given to statements mentioned by individual participants. |
