# Supplementary material for: Caregivers and multidisciplinary team members’ perspectives on shared decision making in Duchenne muscular dystrophy: A qualitative study
Source: Orphanet J Rare Dis. 2025 Mar 10;20:113. doi: 10.1186/s13023-025-03555-0 (PMC11895160; doi:10.1186/s13023-025-03555-0)
Supplement: Supplementary file 2 — Supplementary Material 2 [file 13023_2025_3555_MOESM2_ESM.docx]

**Appendix 2: Final code book for the analysis of the interviews.**

| **Code** | **Description** | **Inclusion criteria** | **Exclusion criteria** | **Example** |
| --- | --- | --- | --- | --- |
| ***Shared decision making*** | | | | |
| Knowledge | Previous knowledge on shared decision making | Whether or not participants have heard of shared decision making and how they define the process | The source of information regarding shared decision making or additional information the participant would like to acquire about this process | *"I have not heard of it yet."* |
| Perception | General opinion and perception of shared decision making | Opinions and perceptions that participants have regarding shared decision making and the consequences thereof | Perceptions on how shared decision making is currently implemented | *"I think this is an important process to improve treatment adherence."* |
| Benefits | Benefits of shared decision making | The participant lists possible benefits of shared decision making | Drawbacks, barriers, or facilitators of shared decision making | *"I definitely see clear advantages: that the patient is well-informed and able to decide for themselves."* |
| Drawbacks | Drawbacks of shared decision making | The participant lists possible drawbacks of shared decision making | Benefits, barriers, or facilitators of shared decision making | *"I think disadvantages can arise when a patient doesn't want to make a decision themselves, and as a healthcare provider, you make the decision for them based on what you professionally judge to be the best choice. They might later blame you for that, and that can definitely be a drawback."* |
| Barriers | Barriers of shared decision making | The participant lists possible barriers of shared decision making | Benefits, drawbacks, or facilitators of shared decision making | *"Requires energy from both parents and doctors."* |
| Facilitators | Facilitators of shared decision making | The participant lists possible facilitators of shared decision making | Benefits, drawbacks, or barriers of shared decision making | *"A strong team with different expertise that can guide parents, each with a different perspective and view on the treatment possibilities."* |
| Current implementation | Perceptions on how shared decision making is currently implemented | The participant lists experiences and insights on how shared decision making was applied during previous consultations or how this is applied in practice including the current role of all members of the multidisciplinary team | General perception of shared decision making | *"I really do think that's how I do it or try to do it. I don't have that theory in my mind so much. But I do think I approach it that way."* |
| ***New and emerging treatments*** | | | | |
| Knowledge | All previous knowledge on new and emerging treatments | Amount of knowledge that participants have on new and emerging treatments, ranging from basic to extensive | The source of information about or additional information the participant would like to acquire | *"I have a good understanding of most things, not how every molecule works, but well-informed about the variety that exists."* |
| Source | The source of the previous knowledge on new and emerging treatments | The places, events, or persons where the participant gained information on new and emerging treatments such the treating physician, etc. | The difficulty of finding this information or the content of the information received from the source | *"We do receive regular updates here, like a lesson from our doctor, so they are indeed making an effort to keep our team well-informed about the new matters"* |
| Questions | Questions regarding new and emerging treatments | Expression of the need/questions for additional information on new and emerging treatments | Expression of feelings towards new and emerging treatments | *"From what age will we be able to apply the therapies, or are there ideal time frames to start using them?"* |
| Future role in shared decision making | Perception of future role in SDM | Participants perceptions on what role they could have in the future when these new and emerging treatments become commercially available | Participants perceptions on current role in shared decision making | *"If and when the therapy becomes available, we will naturally conduct all the counseling and related procedures, right? We will inform the individuals, then we will provide the treatments to them, and simply initiate them"* |
| ***Patient decision aids*** | | | | |
| Knowledge | All previous knowledge on patient decision aids | Whether or not participants have heard of a patient decision aid and how they define it | The source of that information or additional information the participant would like to acquire | *"I have not heard of it yet."* |
| Perception | General opinion and perception of a patient decision aid | Opinions and perceptions regarding a patient decision aid for Duchenne muscular dystrophy | Benefits or drawbacks on a patient decision aid for Duchenne muscular dystrophy | *" Such a tool can certainly help. However, I believe it's crucial to exercise caution in how the questions are framed."* |
| Benefits | Benefits of a patient decision aid | The participant lists possible benefits of a patient decision aid | Drawbacks or perceptions of a patient decision aid for Duchenne muscular dystrophy | *"It can be convenient for adult patients to view at home, allowing them to discuss what aspects they consider significant for their child."* |
| Drawbacks | Drawbacks of a patient decision aid | The participant lists possible drawbacks of a patient decision aid | Benefits or perceptions of a patient decision aid for Duchenne muscular dystrophy | *"The information provided can become outdated quickly."* |
| Structure | Structure regarding a patient decision aid for Duchenne muscular dystrophy | Participants preference in structure regarding patient decision aid e.g. Information aspects | Participants preference in format, content, lay-out, or language regarding a patient decision aid | *"Simple and easy to understand."* |
| Format | Format regarding a patient decision aid for Duchenne muscular dystrophy | Participants preference in format regarding patient decision aid e.g. Information aspects | Participants preference in structure, content, lay-out, or language regarding a patient decision aid | *"Both digitally and on paper."* |
| Content | Content regarding a patient decision aid for Duchenne muscular dystrophy | Participants preference in content regarding patient decision aid e.g. Information aspects | Participants preference in structure, format, lay-out, or language regarding a patient decision aid for Duchenne muscular dystrophy | *"I think it should be as clear and comprehensive as possible so that the patient is informed about all aspects, including the physical aspects, but also the emotional or the consequences associated with that therapy in daily life."* |
| Lay-out | Lay out regarding a patient decision aid for Duchenne muscular dystrophy | Participants preference in lay out regarding patient decision aid | Participants preference in structure, format, content, or language regarding a patient decision aid for Duchenne muscular dystrophy | *'I think visual aspects are important"* |
| Timing | Timing regarding a patient decision aid for Duchenne muscular dystrophy | Participants perception on the ideal timing for the implementation of a patient decision aid | Benefits or drawbacks on a patient decision aid for Duchenne muscular dystrophy | *'"I believe it's best before the consultation, but after the initial diagnostic conversation."* |
| Language | Content language of a patient decision aid for Duchenne muscular dystrophy | Participants preference in language regarding patient decision aid | Participants preference in structure, format, content, or lay-out regarding a patient decision aid for Duchenne muscular dystrophy | *"I believe that, in any case, when making serious decisions, it should be in one's native language."* |
| Future use | Perceptions of future use of a patient decision aid for Duchenne muscular dystrophy | Participants interest on implementing a patient decision aid and how a patient decision aid can be implemented in the future when available. | Benefits or drawbacks on a patient decision aid for Duchenne muscular dystrophy | *"I would definitely consider that a useful format, a kind of guide to ensure nothing is overlooked and to work in a structured manner"* |
